# Common Coagulopathies Associated With COVID-19 Patients

**DOI:** 10.7759/cureus.38067

**Published:** 2023-04-24

**Authors:** Vinish Gupta, Sourya Acharya, Akshunna Keerti

**Affiliations:** 1 Department of Medicine, Jawaharlal Nehru Medical College, Datta Meghe Institute of Medical Sciences, Wardha, IND

**Keywords:** disseminated intravascular coagulation, thrombosis, thrombocytopenia, d-dimer, coagulopathy, sars-cov-2

## Abstract

The coronavirus disease 2019 (COVID-19) outbreak, which first appeared in the Chinese province of Hubei city of Wuhan, has been spreading internationally since December 2019. The World Health Organization (WHO) declared the coronavirus illness from 2019 to be a pandemic on March 11, 2020. Patients hospitalised with severe coronavirus or comorbid conditions (like cardiovascular disease and obesity) are linked to a worse prognosis. The rise in D-dimer and its relationship to prognosis are the most often documented aberrations in coagulation/fibrinolysis in COVID-19. However, the D-dimer assessment's utility is not limitless. Since the coagulation/fibrinolytic state might occasionally change over a short period of time, routine exams are also advantageous in understanding the relevance of the inquiry. Both thrombotic and hemorrhagic diseases should be taken into consideration, despite the fact that the pathophysiology of disseminated intravascular coagulation (DIC) linked with coronavirus disease 19 differs significantly from that of septic disseminated intravascular coagulation. Coagulation as well as fibrinolysis indicators are used to make the diagnosis of COVID-19 thrombosis, which encompasses both macro- and micro-thrombosis. Compared to bacterial-sepsis-associated coagulopathy/DIC, COVID-19 has a lower prevalence of prolonged prothrombin time, activated partial thromboplastin time, and decreased antithrombin activity. However, the causes of coagulopathy remain poorly understood. Hypoxia, endothelial injury, dysregulated immunological responses mediated by inflammatory cytokines, and lymphocyte cell death are thought to be implicated. While blood loss tends to be rare, it is uncertain if COVID-19 suffers from thrombosis or whether the current recommendations for regular venous thromboembolic dose are appropriate. It is important to decide on the COVID-19 therapy phases. Antiviral therapy, cytokine storm therapy, and thrombosis therapy are the steps. Future advancements are predicted, such as a therapy that combines heparin and nafamostat.

## Introduction and background

Coronavirus disease 2019 (COVID-19), a syndrome brought on by severe acute coronavirus syndrome 2 (SARS-CoV-2), causes severe acute respiratory symptoms. 80% of persons with the condition are asymptomatic or exhibit only very mild symptoms, compared to 20% who develop significant illness and 2% to 5% who die [1]. The geriatric group and patients with comorbidities (including cardiovascular disease, hypertension, diabetes mellitus, and obesity) are more prone to getting more serious diseases. Males are known to contract infections more frequently than females. But even in young people and healthy individuals, serious illness can arise, and many aspects of what affects the outcomes are still unclear [2]. There can also be some inherited variables at play. Individuals with the O blood group have a relatively lower chance of acquiring COVID-19 as compared to non-O group individuals, and there have been reports of gender differences in the immune responses to this virus [3].

The primary cause of mortality is respiratory failure. Multiple organ failure, thrombosis, disseminated intravascular coagulation (DIC), activation of the coagulation system in association with excessive immune/inflammatory reactions (representing the so-called cytokine storm), and more are other issues [4]. Particularly, thrombosis and DIC have the potential to significantly exacerbate the disease.

In addition to mild thrombocytopenia and coagulation problems, patients with COVID-19 pneumonia commonly have elevated levels of fibrinogen and D-dimer. Elevated D-dimer levels have been associated with a higher death rate [5]. In a subgroup of COVID-19 people, prothrombin time/international normalised ratio (PT/INR) and activated partial thromboplastin clotting time (aPTT) might be abnormally short [6]. The shorter aPTT is usually linked to increased Factor VIII as an acute-phase response (FVIII) [6]. In more severely ill individuals, a syndrome that resembles DIC with relatively mild PT and aPTT prolongation (although fibrinogen tends to stay normal/elevated) may develop [7].

By the standards of the International Society of Thrombosis and Haemostasis (ISTH) [7], DIC is still regarded as having abnormal D-dimer levels even though D-dimer parameters are markedly increased to any anomalies detected in the PT/INR [8], aPTT, fibrinogen, or platelet. In contrast to the typical pattern of DIC brought on by bacterial sepsis or trauma, coronavirus exhibits minor aPTT and/or PT prolongation, mild thrombocytopenia (a platelet count of 100-150 109/L), sporadic hypofibrinogenemia, as well as sporadic laboratory evidence of hyperfibrinolysis [9]. The term "COVID-19-associated coagulopathy" refers to this spectrum of coagulation disorders [10]. The literature has identified three stages of coronavirus-related coagulopathy: stage 1, which is characterised by elevated D-dimer; stage 2, which is characterised by elevated D-dimer as well as a mild increase in PT/INR and aPTT as well as mild thrombocytopenia; and stage 3, which is characterised by critical illness and laboratory findings that progress towards classic disseminated intravascular coagulation. [11].

## Review

Methodology

**Figure 1:**
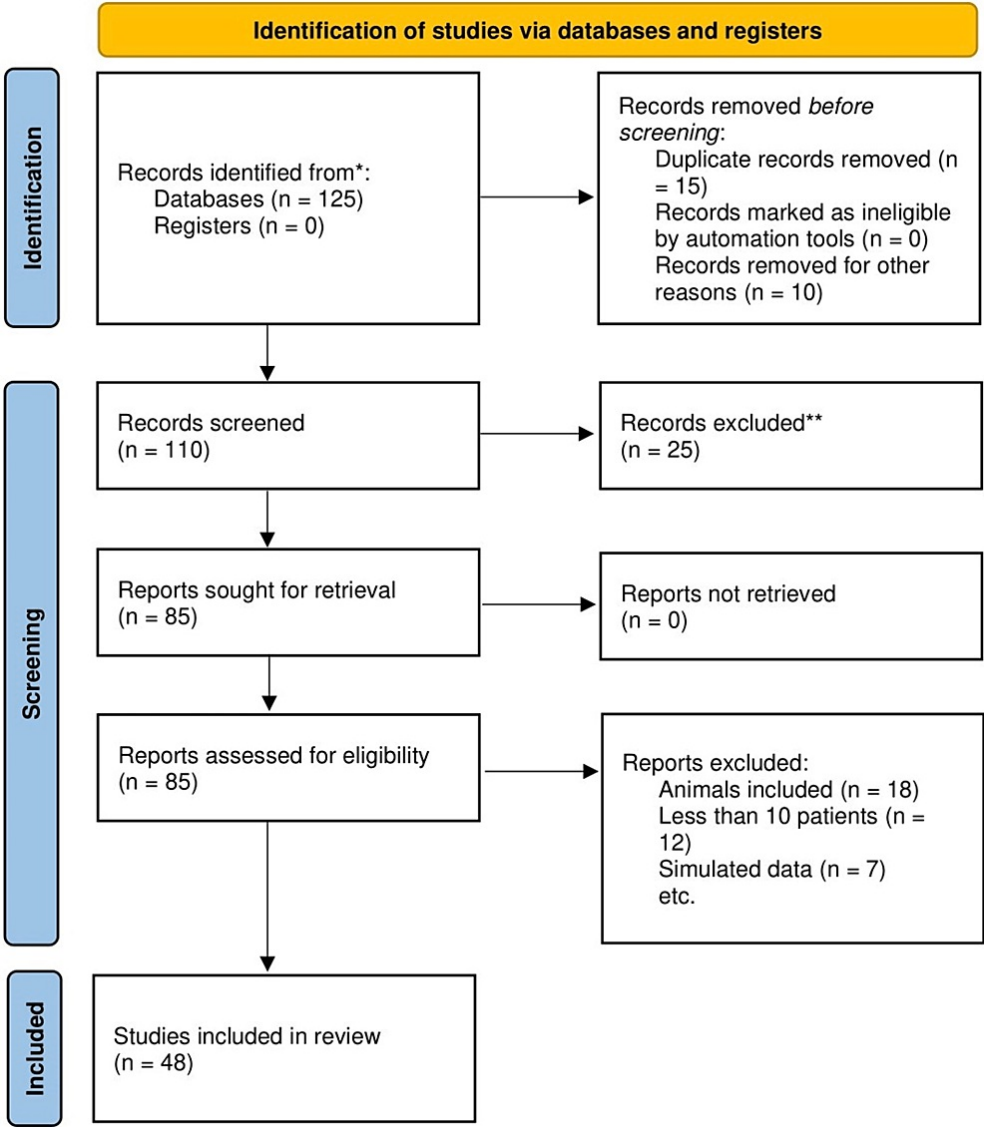
PRISMA flow diagram 15 records were removed due to duplication. 25 records were excluded due to a lack of correlation between coagulopathy and COVID-19

Google Scholar, Science Direct, PubMed, and Google Search were all employed in the comprehensive electronic literature search for this review. The terms "SARS-CoV-2," "Coagulopathy," "D-dimer," "Thrombocytopenia," "Thrombosis," and "Disseminated intravascular coagulation" were used both individually and together. All relevant studies, including reviews, meta-analyses, organisational submissions, and original research, were carefully arranged. Even though highly regarded earlier studies were regularly mentioned, the most recent study was given preference. The flow diagram for PRISMA is shown in Figure 1 below.

COVID-19 associated coagulopathy

A coagulation profile, which includes the measurements of D-dimer, partial thromboplastin, partial thromboplastin time, and platelet count, should be carried out in hospitalised patients with suspected or confirmed COVID-19 [12]. These indicators may change 4-10 days after admission to the hospital or 7-11 days after the onset of symptoms. D-dimer, prothrombin time, and platelet count should all be repeated in patients with severe COVID-19 at least once every two to three days.

A prolonged PT, thrombocytopenia, and a high D-dimer are all signs of DIC [13]; however, the disease does not present the same way as sepsis, where the thrombocytopenia is much more severe and the elevation in the D-dimer does not approach the levels seen in patients with coronavirus disease 2019 [14]. In the majority of individuals with severe illness, low-grade DIC and pulmonary thrombotic microangiopathy-which are thought to be related to COVID-19-are likely to result in serious organ failure.

One feature of the severe coronavirus 2019 sickness is the emergence of coagulopathy as a part of the systemic inflammatory response syndrome. Hematologic abnormalities in coagulation tests, such as increased D-dimer, prolonged PT, thrombocytopenia, and decreased fibrinogen levels, were seen in 20-50% of COVID-19 patients who were hospitalised [15]. This syndrome is characterised by thrombotic episodes instead of haemorrhagic occurrences (venous thromboembolism [VTE]). In COVID-19 deaths, fibrin and thrombin deposition, mainly in the pulmonary microvasculature, is the primary cause of acute respiratory distress syndrome, coagulopathy, the thrombosis of central lines and catheters, and vascular occlusive events (cerebrovascular events, limb ischemia, etc.) [16]. Furthermore, the hypoxia that develops in severe COVID-19 may exacerbate thrombosis by thickening the blood and initiating a signalling cascade that depends on hypoxia-inducible transcription factors [17].

The pathophysiology of the microcirculatory changes brought on by SARS-CoV-2 infection involves a specific kind of endotheliopathy. The sepsis-induced coagulopathy (SIC), which includes impaired fibrinolysis and elevated thrombin generation, is analogous to this endotheliopathy. The angiotensin-converting enzyme-2 (ACE-2) receptor on endothelial cells serves as the viral adhesion receptor, and viral replication results in inflammatory cell infiltration, endothelial death, and microvascular prothrombotic events [18]. Mononuclear and polymorphonuclear cell infiltration, viral inclusions inside endothelial cells, and signs of endothelial mortality have all been found during post-mortem investigation of SARS-Cov-2 infection. The clinical effects that COVID-19 patients experience as a result of microcirculatory dysfunction are therefore important. Additional anomalies that may be applicable in coagulopathy include low fibrinogen, increased Lactate dehydrogenase (LDH), and, in certain cases, substantially raised blood ferritin values.

The procoagulant response during the acute phase of COVID-19 infection is another crucial aspect of the disease. Acute phase reactants such Factor VIII, von Willebrand factor (vWF), and fibrinogen are linked to a higher risk of thrombosis and have an impact on fibrinogen levels. Inflammatory cytokines that are produced in greater amounts during the latter stages of the disease include tumour necrosis factor and interleukins, notably interleukin-1 (IL-1) and interleukin-6 (IL-6) [18]. Thrombin is produced by macrophages, and IL-6-induced tissue factor expression causes coagulation to start [19]. The main mediators of the endogenous coagulation cascade's inhibition are tumour necrosis factor and IL-1. A cytokine storm with high proinflammatory cytokines and chemokines levels may occur in a group of very ill individuals, victims of COVID-19 [20].

When plasmin breaks down stabilised fibrin polymer [21], which is fibrin crosslinked with factor XIII, D-dimer is produced in the blood. The body produces the thrombus as a result of coagulation activation, which is then broken down by fibrinolytic activation. Several publications have observed the connection between elevated D-dimer levels and severity [22]. However, if a considerable amount of thrombus builds up in the body but is not broken down, the increase in D-dimer may be small (indicating the most severe scenario for the body) [23]. Even in dire situations like death, the increase in D-dimer is only marginally perceptible, particularly in the suppressed-fibrinolytic-type disseminated intravascular coagulation brought on by sepsis [24]. As a result, the degree of the D-dimer increase does not always correlate with the severity of the clinical condition [25].

Anticoagulation

If there are other diseases present (such as cardiovascular disease, obesity, sepsis-induced coagulopathy score > 4, high D-dimer (>6 times the normal), C-reactive protein (CRP), troponins, and other symptoms of disseminated intravascular coagulation) [26], hospital mortality for patients with severe COVD-19 can increase to 42%. It has been demonstrated that by stopping the growth of microthrombi and the associated pulmonary coagulopathy and decreasing concurrent inflammation, low molecular weight heparin (LMWH) given as the first anticoagulant treatment in this group significantly increases arterial oxygen pressure/inspired fraction of oxygen (PaO2/FiO2). Mortality was seen to have dropped by 48% and 37% in 7 days and 28 days, respectively [27].

Unless the patient has severe thrombocytopenia (<50,000 mm or active bleeding), anticoagulation should be evaluated for VTE evidence or if the patient is anticoagulated [27]. The drug is selected based on how well the kidneys, liver, platelets, and digestive system are functioning. Parenteral anticoagulation is recommended for critically ill patients since it can be temporarily stopped and does not interact negatively with any of the drugs being tested to treat COVID-19 [28]. Unfractionated heparin exposure via paraclinical and dose adjustments exposes medical personnel, hence LMWH is recommended in critically ill patients [29]. Direct oral anticoagulants provide a number of benefits, including the simplicity of outpatient care and the absence of ongoing monitoring [30]. However, there are hazards associated with their usage, including clinical deterioration and a shortage of reversal drugs at all sites. In patients who will be discharged, direct oral anticoagulants and LMWH should be chosen over routine INR testing [31].

After 4 to 14 days of heparin medication, a 30-50% decrease in platelet count should be regarded as a symptom of thrombocytopenia brought on by heparin. Suspending this anticoagulant medication is necessary because of the aforementioned factors, some of which may also contribute to the limb ischemia seen by COVID-19 patients [32]. In addition to heparins, the synthetic serine protease inhibitor nafamostat mesylate is thought to be a strong inhibitor of the Middle East respiratory syndrome coronavirus (MERS-CoV) infection and the Ebola virus [33]. It also has anticoagulant effects, such as the inhibition of Factor VIIa. This chemical stops cathepsin B from impeding the viral surface glycoprotein's proteolytic digestion [34].

For patients with a history of bleeding who are receiving cardiopulmonary bypass or continuous renal replacement treatment, nafamostat is also licenced as an anticoagulant medication. It has anticoagulant, antifibrinolytic, and antiplatelet properties. It blocks a number of serine proteases generated during the coagulation cascade and the inflammatory process, including activated Factors VIIa and XIIa, kallikrein, thrombin, elements of the complement system, and trypsin [35]. It also blocks tissue-type and urokinase plasminogen activators. When used to prevent blood coagulation during extracorporeal blood circulation, nafamostat should be continuously given at a dosage of 20 to 50 mg/h. It has been determined that nafamostat is a powerful inhibitor of S-mediated membrane fusion [36]. In severe COVID-19 patients where thrombotic problems are frequent, nafamostat's anticoagulant, antiplatelet, and antifibrinolytic properties may be beneficial in addition to its claimed antiviral efficacy. It has been established that nafamostat is a secure and reliable anticoagulant [37]. To prevent bleeding, one must be aware of possible interactions with other anticoagulant therapies that may be used concurrently.

Platelets

In 2003, between 20% and 55% of SARS patients had thrombocytopenia. A later rebound thrombocytosis was also noted. Higher rates of morbidity and mortality were seen in patients who had thrombocytopenia throughout the epidemic [38]. The thrombocytopenia was also connected to MERS [39].

Depending on the severity of the illness, thrombocytopenia, which is commonly mild (counts are frequently between 100 and 150 109/L), is found in 5.4-41.7% of COVID-19 patients. In 58-95% of COVID-19 severe cases, mild thrombocytopenia was seen; individuals with severe illness frequently had platelet counts 23-31 109/L lower than those with non-severe disease [39]. Average platelet counts are found in patients who are ill and have systemic immunological and coagulation activity, which shows a large compensatory platelet production response. Rarely have reports of significant thrombocytopenia in COVID-19.

For a number of reasons, viral infection and thrombocytopenia can coexist. Increased platelet clearance/destruction frequently facilitates the rapid start of thrombocytopenia in response to viral infections, even while hypo proliferative thrombocytopenia is present in the later stages of viral disease. Platelets that have been activated by viral antigen-antibody complexes or inflammatory reactions from the host are removed from circulation more quickly by the reticuloendothelial system [40]. Viruses can also affect megakaryocytes and reduce platelet production.

Platelets are necessary for the body to react to infections and signal inflammation. Platelets may aid in concentrating haemostasis and immune responses against potential infectious agents to block the microbial invasion by combining thrombotic and immunological recruitment capacities. Platelets directly interact with viruses through a variety of receptors, such as Toll-like receptors [41]. Platelets can agglomerate and absorb diseases, but they cannot kill microorganisms. Platelets and the products they make have been seen to either support or suppress viral infection, depending on the particular infection. Platelets' capacity to attract and activate circulating leukocytes on the surface of the endothelium is thought to be the cause of white blood cell diapedesis. The procoagulant effect of viral infections depends on interactions between leukocytes, platelets, and endothelial cells [42]. In viral infections, thrombocytopenia, platelet secretion, and interactions with leukocytes may have either unfavourable or good immunological effects.

While platelets assist in preserving the basal barrier integrity of the alveolar capillaries, they may also play a role in the onset of a number of pulmonary illnesses and syndromes that cause lung injury. In animal models, platelet-leukocyte aggregates and platelet-endothelial interactions appear to be associated with acute lung injury brought on by physical/chemical damage and influenza infection, respectively [43]. For instance, during dengue infection, platelet-derived IL-1 increases endothelial permeability.

Antiplatelet agents

Although there is no randomised study to support this, antiplatelet medicines may play a role in COVID-19 disease [44]. Particularly for people with mild thrombocytopenia, such treatment would have an increased risk of bleeding. Treatments for coronavirus, such as lopinavir/ritonavir, remdesivir, bevacizumab, tocilizumab, sarilumab, fingolimod, chloroquine/hydroxychloroquine, interferon, and azithromycin, may interact harmfully with antiplatelets [44].

Discussion

The indication suggests a combination of localised pulmonary platelet consumption, low-grade disseminated intravascular coagulation (rarely meeting the ISTH DIC criteria), and variably thrombotic microangiopathy as the pathogenesis of coronavirus coagulopathy. In post-mortem studies, higher amounts of vWF and soluble thrombomodulin are indicative of activated or injured endothelium [45]. Ultra-large vWF multimers that interact with platelets and can cause platelet activation, microthrombi, and platelet consumption are expected to be produced by damaged endothelium. Increased activation of the endothelium and/or circulating platelets is suggested by elevated levels of soluble P-selectin and platelet flow cytometric investigations in COVID-19 patients [46]. The SARS-CoV-2 Spike protein directly activates platelets, according to recent studies. There is a link between severe COVID-19 illness and more platelet activation and platelet-monocyte aggregation. Platelets from seriously unwell individuals with COVID-19 can increase monocyte tissue factor (TF) expression (under the control of P-selectin and IIb/3), which may exacerbate inflammation and hypercoagulability in these patients [47].

Findings reveal that severe cases of thrombotic coagulation disorder are very common, despite the fact that our understanding of coagulopathy in COVID-19 is still in its infancy. The prevalence of thrombocytopenia is less common than septic shock, despite the fact that the D-dimer is a more accurate indication of the severity and more sensitive than other coagulation markers [48]. Contrary to thrombotic events, bleeding problems are far less prevalent in COVID-19, hence anticoagulant therapy is not required.

## Conclusions

There are still many ways in which the pathophysiology of COVID-19 has to be fully described. Laboratory haematology research is still being done to better understand pathophysiology. Despite its limitations in determining the reason behind the coagulation anomalies seen by COVID-19, D-dimer is an excellent diagnostic tool. It is anticipated that adding markers like thrombin-antithrombin complex (TAT), plasmin-α2 plasmin complex (PIC), and soluble fibrin (SF) will make the pathological condition clearer.

The COVID-19 pandemic may have a variety of effects on thrombotic or thromboembolic disease prevention and therapy. First, think about how the cytokine storm that produces COVID-19 could be impacted directly or indirectly. According to the most recent national and international recommendations, each institution should adhere to the protocols for thromboprophylaxis, anticoagulation, and other considerations for managing coagulopathy and bleeding.
